# Complementary role of computed tomography angiography with fixed perfusion defects on SPECT myocardial perfusion imaging

**DOI:** 10.1186/s41824-021-00124-z

**Published:** 2022-02-01

**Authors:** Rami M. Abazid, Yasmine T. Sallam, Jonathan G. Romsa, James C. Warrington, Cigdem Akincioglu, Sabe De, Nikolaos Tzemos, William C. Vezina

**Affiliations:** 1 Division of Nuclear Medicine, Section of Cardiac Hybrid Imaging, Victoria Hospital, London Health Sciences Centre, 800 Commissioners Road East, PO Box 5010, London, ON N6A 5W9 Canada; 2 Division of Medical Imaging, Northern Ontario School of Medicine, Sudbury, ON Canada; 3 Division of Cardiology, Department of Medicine, London Health Sciences Centre, London, ON Canada

**Keywords:** Perfusion defects, SPECT, Coronary CT angiography

## Abstract

**Purpose:**

We present this case series exploring the complementary role of coronary computed tomography angiography (CCTA) to SPECT myocardial perfusion imaging (MPI) in the detection of myocardial necrosis.

**Methods:**

A cardiac hybrid imaging database search identified 144 patients with a previous history of ST-segment elevation myocardial infarction treated with coronary revascularization. CCTA and MPI scans were evaluated to determine whether CCTA had an added value to MPI in detecting myocardial necrosis.

**Results:**

Five patients with patent stents and/or bypass grafts and both fixed perfusion defects on MPI and sub-endocardial hypo-perfusion on CCTA were identified. The extent and location of the perfusion defects were closely correlated between the CCTA and SPECT MPI images.

**Conclusion:**

In this series, CCTA and SPECT MPI were found to play a complementary role in the assessment of fixed perfusion defect, with CCTA adding specificity to the diagnosis of myocardial necrosis.

## Introduction

Fixed perfusion defect (FPD) is a perfusion abnormality that is detected on both rest and stress SPECT MPI scans of the identical location, size and severity (Garcia et al. 2020; Busch et al. 2011). The presence of FPD is suggestive nonviable myocardial tissue. However, other causes for FPD include hibernating/viable myocardium and attenuation artifacts. The differentiation between infarct/scar and viable myocardium can be made with Tl-201 single-photon emission computed tomography (SPECT) as well as Fluorodeoxyglucose-positron emission tomography (FDG-PET). Late gadolinium enhancement with cardiac magnetic resonance imaging is a specific finding of myocardial scar. On the other hand, the presence of contractile reserve with dobutamine echocardiography is regarded as a specific echocardiographic sign of a viable myocardium (Garcia et al. 2020). To date, few studies evaluated the diagnostic accuracy of first-pass CT myocardial perfusion with late-phase CT imaging (delay enhancement) in the detection of myocardial scar. Additionally, no definite criteria exist to identify non-viable myocardium with coronary computed tomography angiography (CCTA) without using the delay enhancement scan (Garcia et al. 2020; Busch et al. 2011).

The purpose of this analysis is to explore whether CCTA has added value to SPECT MPI in diagnosing nonviable myocardium.

## Materials and methods

We screened 2364 patients with atypical chest pain who were referred for combined cardiac hybrid anatomic and functional imaging (CCTA and 2-day Rest/Stress SPECT myocardial perfusion imaging (MPI)) in the period between January 2014 and January 2018. All registry patients signed consents to be imaged into a registry to be imaged with CCTA and rest/stress SPECT-MPI and agreed to subsequent follow-up. This registry was approved by the local ethical committee.

A two-day rest/stress protocol with Tc99m Sestamibi was employed for all patients. SPECT MPI imaging was performed on a cadmium–zinc–telluride solid-state detector (CZT) gamma camera with CT attenuation correction. The rest SPECT-MPI study and the CCTA scan were done on the same day. The stress SPECT MPI scan was performed the following day. Physically capable patients were stressed on the treadmill, while pharmacologic stress with dipyridamole ± exercise was performed in patients with limited exercise capacity.

CCTA was performed after oral pre-medication with a goal of reducing the heart rate to less than 60 beats per minute. A 64-slice CT scanner was used for the CCTA within a few hours of the rest-SPECT scan. A timing bolus technique was employed to determine the delay time used to trigger the CCTA acquisition. Late-enhancement CT imaging was not performed as part of this protocol.

**Fig. 1:**
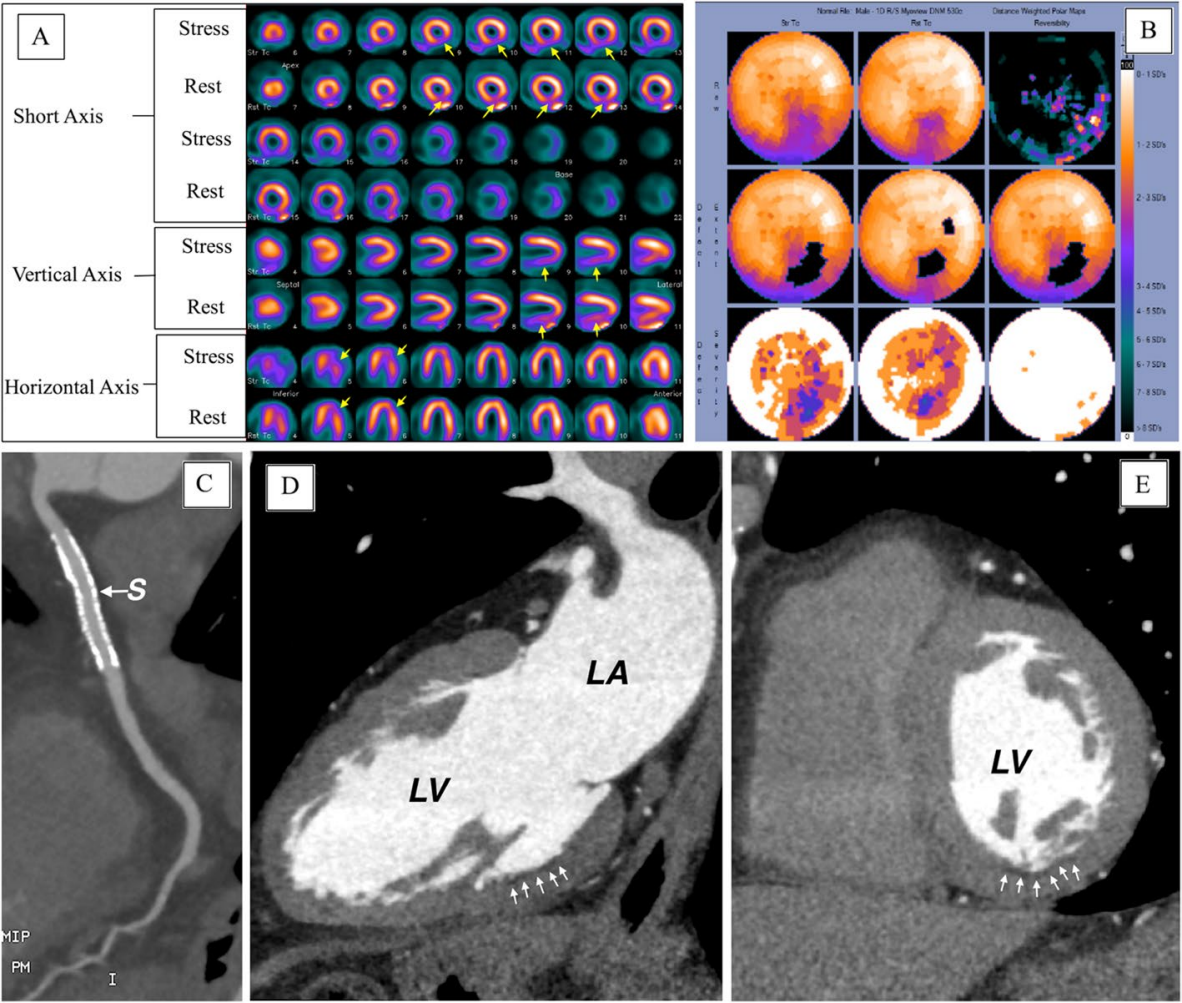
**A** SPECT images, **B** polar maps at stress (upper panel) and rest (lower panel), **C**–**E** computed tomographic angiography images. LV, left ventricle; LA, left atrium

**Fig. 2:**
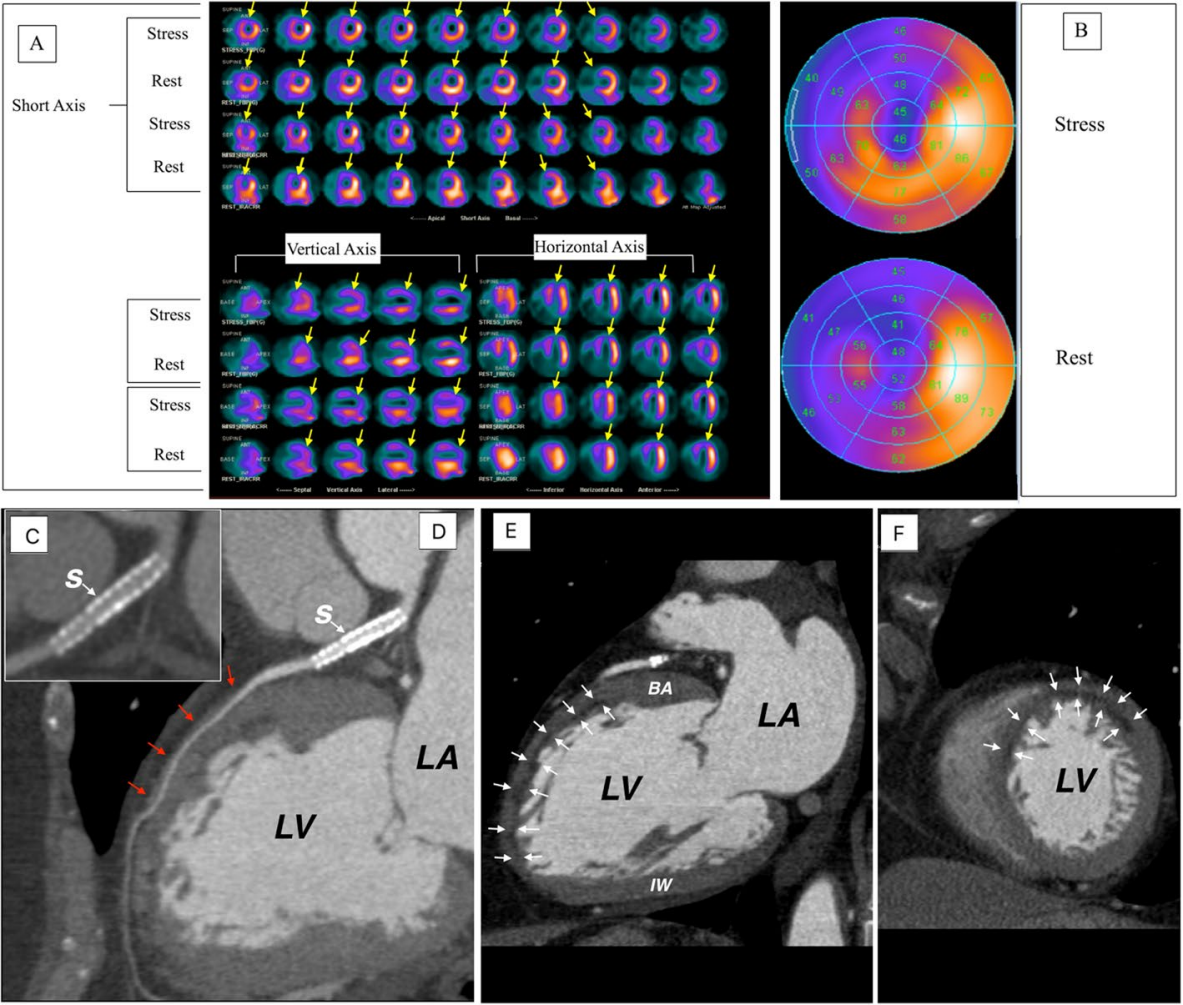
**A** SPECT images, **B** polar maps at stress (upper panel) and rest (lower panel), **C**–**F** computed tomographic angiography images. LV, left ventricle; LA, left atrium; BA, basal anterior wall; IW, inferior wall

**Fig. 3:**
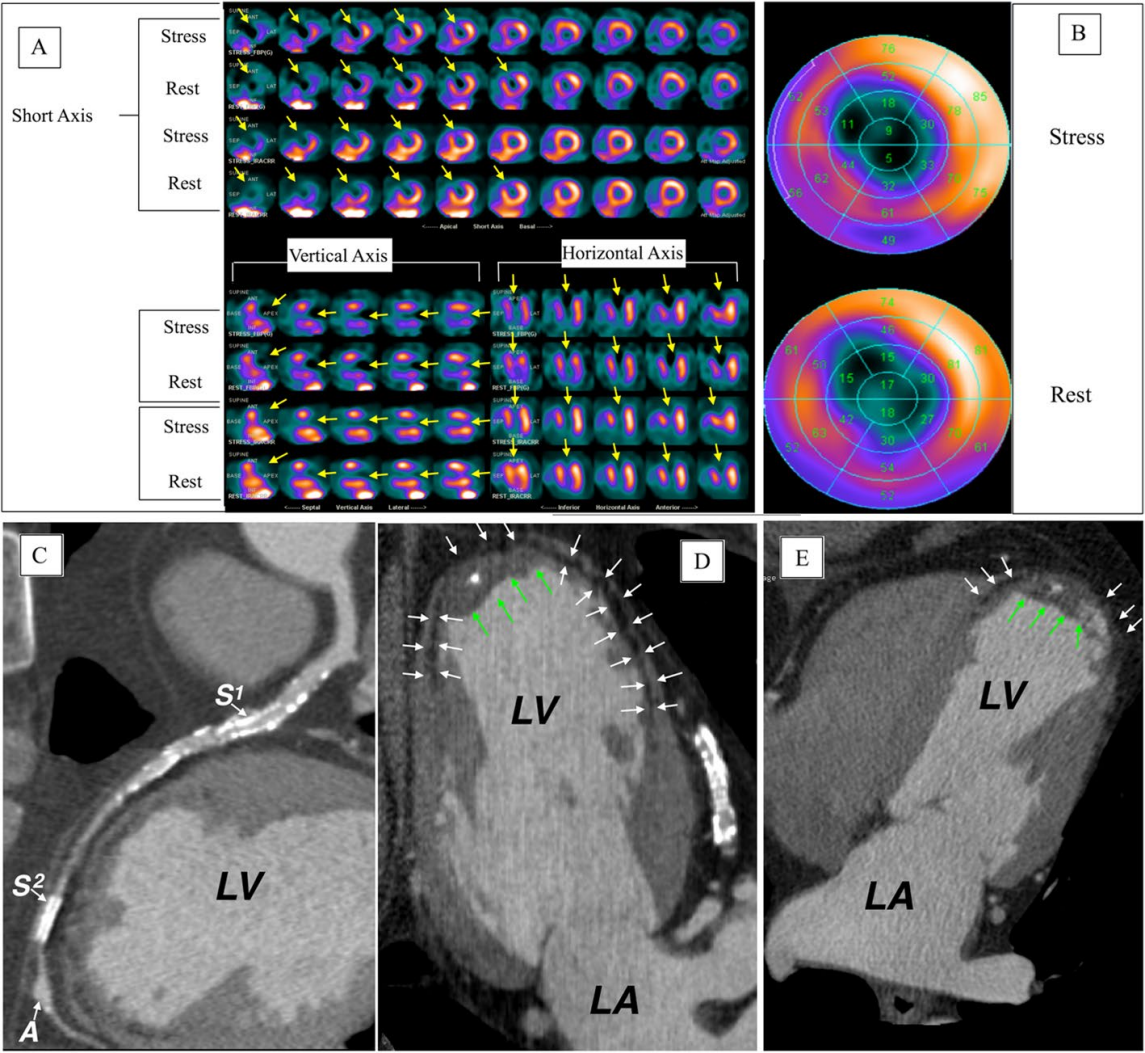
**A** SPECT images, **B** polar maps at stress (upper panel) and rest (lower panel), **C**–**E** computed tomographic angiography images. LV, left ventricle; LA, left atrium

**Fig. 4:**
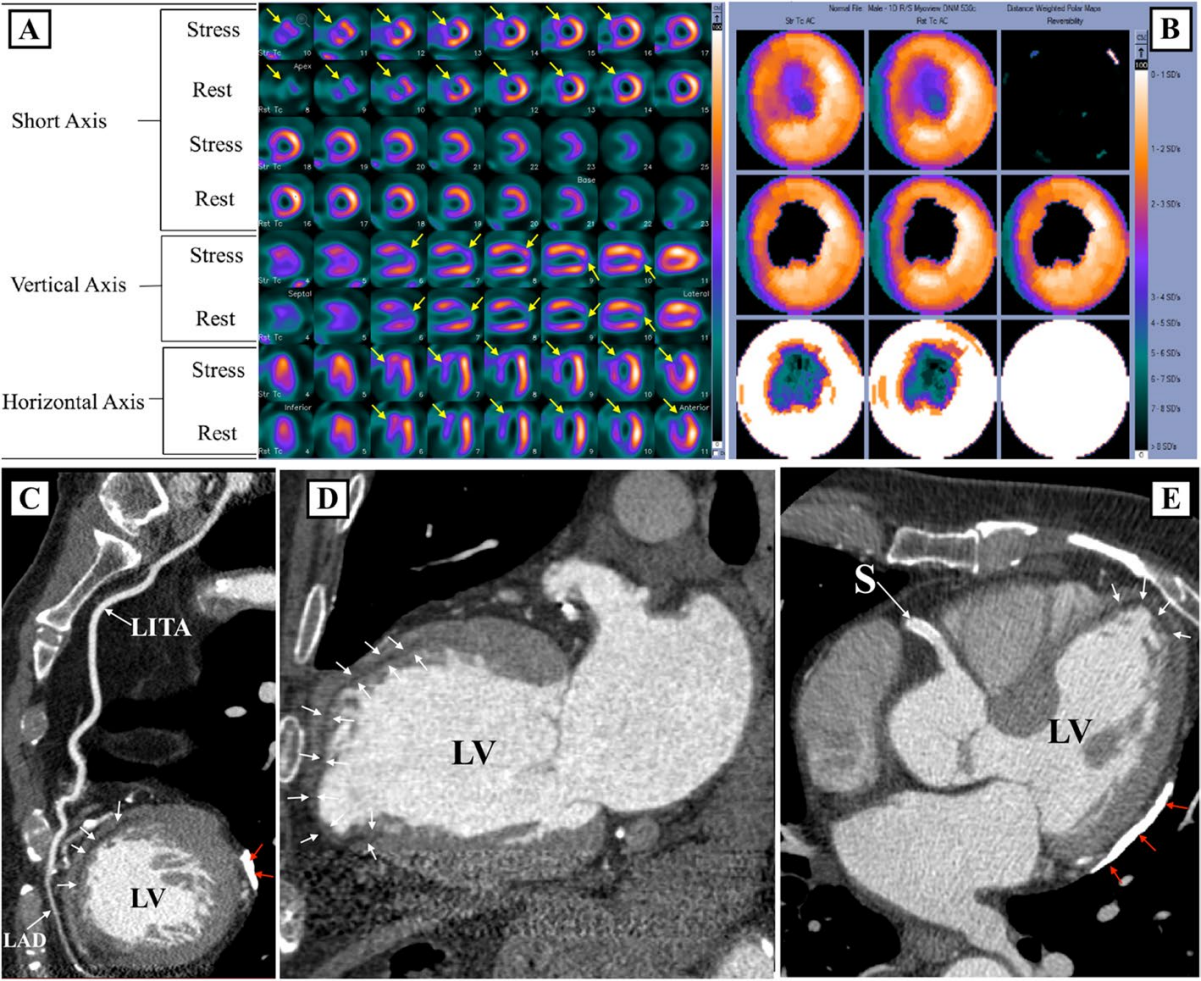
**A**SPECT images, **B** polar maps at stress (upper panel) and rest (lower panel), **C**–**E** computed tomographic angiography images. LAD, left anterior descending artery; LITA, left anterior thoracic artery; LV, left ventricle; S, stent

**Fig. 5:**
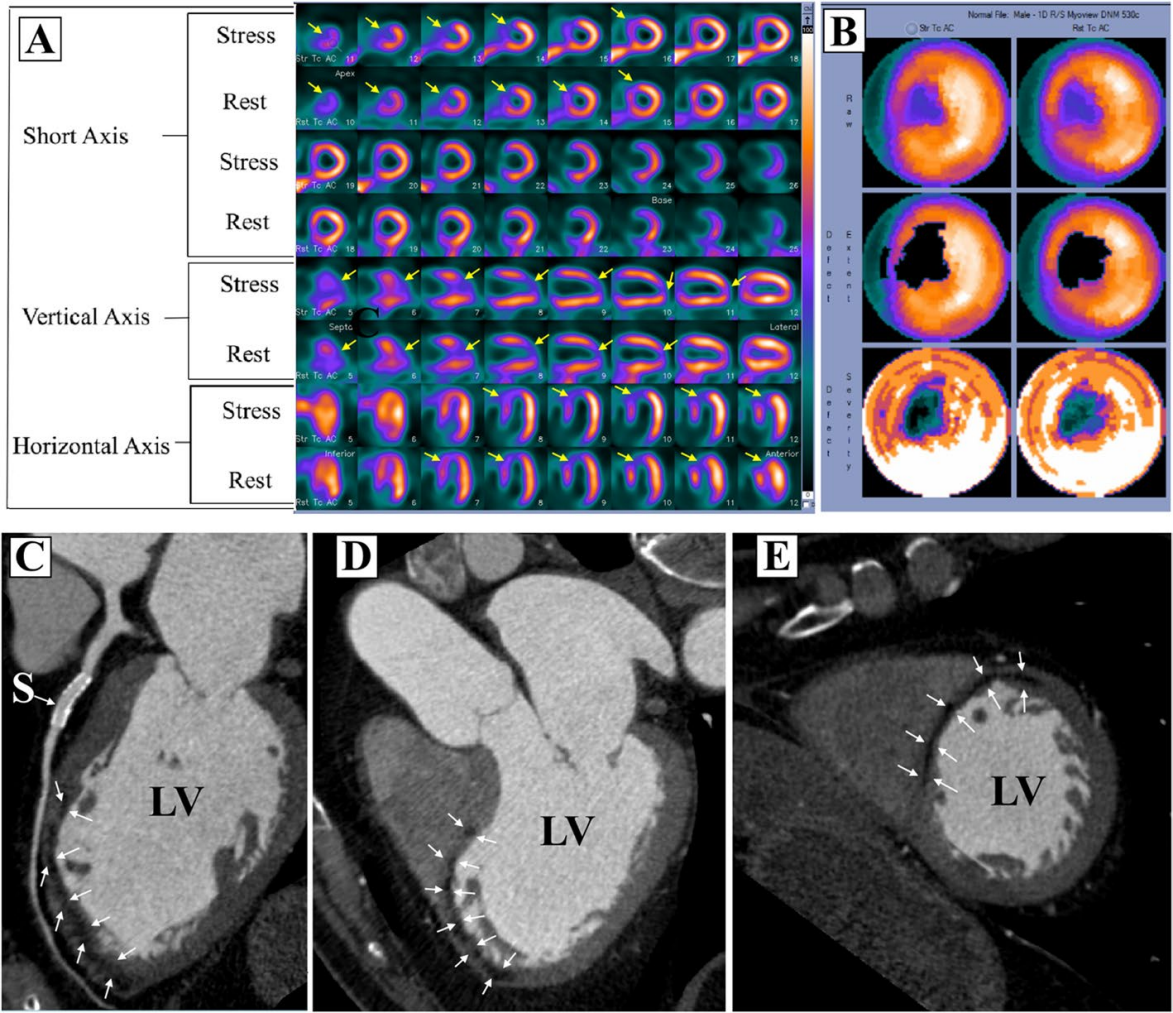
**A** SPECT images, **B** polar maps at stress (upper panel) and rest (lower panel), **C**–**E** computed tomographic angiography images. LV, left ventricle; S, stent

A subgroup of patients (*N* = 144) with a history of ST-segment elevation myocardial infarction (STEMI) who underwent revascularization with either percutaneous coronary intervention (PCI) or coronary artery bypass surgery were reviewed. From that subgroup, we report five patients with both FPD on SPECT-MPI scans and hypo-enhancement on CCTA scans, Figs. 1, 2, 3, 4 and 5. All 5 patients had patents stents/grafts.

## Case series

### Case-1

A 66-year-old male with history of inferior STEMI treated with PCI to the right coronary artery. Echocardiography showed LVEF of 60% with no regional wall motion abnormalities. Six years later MPI (Fig. 1A, B) shows a small fixed defect (< 25% of the wall thickness) at the mid infero-lateral wall segment (yellow arrows) suggestive of attenuation artifact vs a perfusion defect. CCTA images (Fig. 1C–E) show a patent stent (S) with no obstructive lesions and localized mid infero-lateral sub-endocardial hypo-perfusion (white arrows) supporting the MPI finding of a true FPD (myocardial necrosis rather than attenuation artifact).

### Case-2

A 78-year-old female with history of anterior STEMI treated with PCI to the left anterior descending artery (LAD). Echocardiography showed LVEF of 50% with mild hypo-kinesis of the anterior wall. Two years later MPI (Fig. 2A, B) shows a large fixed defect in the anterior wall (yellow arrows) with small area of a peri-infarct ischemia. CCTA (Fig. 2C–F) shows a widely patent stent, mid LAD muscular bridge (red arrows) and diffuse sub-endocardial hypo-perfusion (white arrows) at the anterior and antero-septal wall involving < 50% of the wall thickness. These segments appear thinner than the posterior wall and the basal anterior wall. CCTA supports MPI finding of a fixed defect likely due to endocardial scar after coronary revascularization secondary to STEMI.

### Case-3

A 67-year-old male with history of anterior STEMI treated with PCI to LAD. Echocardiography showed LVEF of 30% with akinetic anterior, anterior-septum and apical segments. Four years later MPI (Fig. 3A, B), shows large severe fixed defect in mid and apical anterior, apex and the adjacent apical segments of the lateral and inferior wall (yellow arrows). CCTA (Fig. 3C–E) shows patent proximal stent (S1) with no focal obstructions, the distal stent (S2) cannot be assessed. Beyond the distal stent there is small LAD aneurysm (A). CCTA also shows a diffuse sub-endocardial hypo-perfusion (white arrows) at the anterior wall and apex involving > 75% of the wall thickness, apical LV aneurysm, thinned out anterior wall and apex and apical LV thrombus (green arrows) with focal calcification.

### Case-4

A 75-year-old male with history of anterior STEMI at age 46 years which was treated with medical therapy. Fifteen years later he underwent hybrid coronary revascularization [robotic-assisted minimally invasive coronary artery bypass surgery of the left internal thoracic artery (LITA) to the LAD, and PCI to RCA]. Echocardiography showed LVEF of 42% with hypo-kinesis of the anterior-septum, apical anterior wall segments and the apex. MPI (Fig. 4A, B), shows a large-sized, moderate to severe fixed defect in the mid and apical anterior, anterior-septum and apex (yellow arrows). CCTA (Fig. 4C–E) shows patent LITA graft and diffuse sub-endocardial hypo-perfusion of approximately 50%-75% of the wall thickness (white arrows) at the anterior, anterior-septal segments, and a thinned-out apex. The proximal RCA stent (S) has no focal obstructions. CCTA also shows a pericardial calcification at the lateral aspect of the heart (red arrows).

### Case-5

A 62-year-old male with history of anterior STEMI treated with PCI to LAD. Echocardiography showed LVEF of 48% with septal hypo-kinesis. MPI (Fig. 5A, B), shows large-sized moderate fixed defect in the anterior-septum, apical anterior wall and apex (yellow arrows). CCTA (Fig. 5C–E) shows patent stent (S) with no focal obstructions and diffuse sub-endocardial hypo-perfusion of 50–75% of the wall thickness (white arrows) at the septum and part of the anterior. These segments appear thinner than the posterior wall and the basal anterior wall.

## Discussion

Myocardial necrosis can be identified with different imaging modalities. It appears as FPD with cardiac SPECT and PET MPI imaging. Dobutamine stress echocardiography has a specificity of 76% and sensitivity of 81% for the detection of viable myocardium and the contractile reserve (Garcia et al. 2020). With cardiac magnetic resonance imaging, the presence of sub-endocardial late gadolinium enhancement has a sensitivity of 95% in diagnosing non-viable myocardium (Garcia et al. 2020).

Myocardial necrosis can also be detected with CCTA as a sub-endocardial hypo-enhancement. There are few reports to have investigated the diagnostic accuracy of hypo-enhancement on dynamic CT perfusion in the diagnosis of myocardial infarction (Busch et al. 2011).

An FPD on SPECT-MPI can result in a diagnostic dilemma as to whether the defect represents hibernating myocardium/repetitive stunning or scar, possibly necessitating additional viability imaging (Dilsizian 2021) or less likely an attenuation artifact. Moreover, when an FPD accompanies a reversible defect, anatomical information with invasive coronary angiography might be required.

In this study, patients were selected with revascularization and *patent* stent/graft on CCTA to support the presumption that the SPECT findings represent a true FPD related to myocardial necrosis, and not hibernating myocardium. In general, CCTA is not recommended in the analysis of stents due to limited diagnostic accuracy, particularly with small stent diameters. However, when CCTA is combined with SPECT MPI in a hybrid cardiac imaging session, many of these limitations can be overcome. CCTA also has non-negligible radiation exposure in real-world practice (Andreini et al. 2020; Hossain et al. 2020). However, newer CT generations enable single heartbeat acquisition and can result extremely low radiation exposure (Kosmala et al. 2019). CZT SPECT is a flexible technology which can dramatically decrease radiation dose by 60%-70% compare to conventional cameras. Thus in the appropriate setting, the radiation dose for hybrid CCTA/MPI studies using CZT cameras is similar to, or less than MPI alone using conventional cameras (Henzlova and Duvall 2020; Schaap et al. 2013).

When indicated, CCTA with rest/stress SPECT can be complementary in the evaluation of patients with prior history of STEMI and revascularization through the detection of myocardial/subendocardial scar, assessment of coronary stents (directly with CCTA or indirectly with SPECT MPI) and non-stented coronary artery segments.

In this report, we illustrate that CCTA is a unique imaging modality, providing both anatomical and perfusion details in patients with previous revascularization. In this dataset of patients with a history of STEMI and patent stents and grafts, FPD on SPECT MPI and subendocardial hypoperfusion on CCTA were found to be largely congruent. The CCTA findings increased the specificity of the SPECT MPI findings for myocardial necrosis as opposed to attenuation artifact or hibernating/viable myocardium. The utilization of a combined hybrid cardiac assessment allowed for a more definitive and comprehensive cardiac assessment. However, our findings cannot be generalized to patients with FPDs and severe coronary artery stenosis, as the hypo-enhancement with CCTA might result from resting ischemia and hibernation. Although promising, further studies enrolling patients without previous revascularization and comparing hypo-enhancement with CCTA with other cardiac viability imaging modalities such as cardiac MRI and FDG-PET are warranted.

## Data Availability

Data sharing is not applicable to this article as the data of the present study are being used in other ongoing study which have not been completed.

## Abbreviations

CCTA: Coronary computed tomography angiography
LAD: Left anterior descending artery
LVEF: Left ventricle ejection fraction
MPI: Myocardial perfusion imaging
PCI: Percutaneous coronary intervention
STEMI: ST-segment elevation myocardial infarction
